# Reducing the Delay Between Stroke Onset and Hospital Arrival: Is It an Achievable Goal?

**DOI:** 10.1161/JAHA.112.002477

**Published:** 2012-06-22

**Authors:** Mathew J. Reeves

**Affiliations:** Department of Epidemiology and Biostatistics, Michigan State University, East Lansing, MI

**Keywords:** acute stroke, pre-hospital delay, thrombolysis

In this issue of the *Journal of the American Heart Association* (*JAHA),* Addo and colleagues^1^ report on prehospital delay by using data from the South London Stroke Registry, a high-quality population-based acute stroke registry that collected data on more than 2000 first-time stroke events over a 9-year period between 2002 and 2010. Among the subset of 1392 out-of-hospital stroke events for which stroke onset-to-arrival (OTA) time data were available, the study found that almost 40% of cases arrived at 1 of the 5 registry hospitals within 3 hours of onset and that the overall median OTA time was 4.7 (interquartile range, 1.5 to 12.7) hours. Unfortunately, data were not presented by study year (ie, 2002 through 2010) to determine if there was any secular improvement in OTA times over this time period. Commendably, 11% of the 1085 ischemic stroke admissions in this study were treated with thrombolysis. In a multivariable logistic regression analysis of prehospital delay (defined as OTA >3 hours), the authors found that black ethnicity, living alone, and nighttime stroke onset were all associated with increased delay, whereas stroke severity was strongly associated with lower odds of delayed arrival. A further multivariable analysis of thrombolysis treatment was undertaken among all 1085 ischemic stroke admissions; the results identified age, ethnicity, and nighttime stroke onset as significant predictors, along with higher stroke severity, which was very strongly related to thrombolysis treatment (presumably because of its direct effects on OTA time). The study also reported on the impact of a 1-year national education campaign (based on the F.A.S.T. [face, arms, speech, and time] stroke assessment criteria) designed to educate the public on stroke signs and symptoms and the benefits of rapid treatment. There was no detectable effect of this campaign on OTA times or thrombolysis treatment.

Given the unique urban location and high-quality methods used in this registry, it is interesting to compare and contrast the findings of this study with the many other previous reports that have covered a diverse range of populations and time periods. The 40% of subjects who arrived within 3 hours and the median OTA time of a little less than 5 hours are well within the range previously reported in a systematic review.^2^ The observation that greater stroke severity was associated with shorter prehospital delay, while stroke onset at night was associated with longer delay is also consistent with previous studies.^3^ Surprisingly, the majority of studies examining the impact of living alone on prehospital delay among stroke patients have shown that living alone is not associated with longer OTA times,^3^ so the finding by Addo and colleagues that living alone was associated with longer delays is an important observation, as is the fact that black patients had longer prehospital delays.

Despite the registry's high level of organization and maturity, it is also important to note that OTA times could not be calculated in 22% (n =454) of the cases. Although there were limited differences in demographic characteristics when cases with missing OTA data were compared to those with OTA data, the fact that stroke severity was markedly lower in the cases with missing OTA data suggests that the registry hospitals did not bother to record onset or arrival times in these patients, either because they had mild or resolving symptoms on arrival or because they had arrived well after the therapeutic window for acute stroke treatment. A recent Get With The Guidelines (GWTG)—Stroke study from the United States examined trends in OTA times and found that 53% of ischemic stroke admissions did not have a documented OTA time.^4^ The cases with missing OTA times had mostly mild symptoms and in all likelihood also arrived after the time window for acute stroke treatments. The point to emphasize here is that having a substantial proportion of patients with missing OTA data, even in high-quality studies such as the South London Stroke Registry, negatively affects our ability to make inferences about the underlying trends and causes of delayed arrival.^5^

Given the limited success of prior mass educational campaigns on reducing OTA times or improving thrombolysis treatment rates,^6^ it is perhaps not surprising that the education campaign described by Addo and colleagues had no statistically significant impact on these 2 outcomes. Although it is clear that education campaigns can improve the public's knowledge and understanding of stroke signs and symptoms, as well as the need for emergency care,^7,8^ their effects on hard clinical outcomes such as arrival times and thrombolysis treatment rates have been unequivocally disappointing.^6^ It has become increasingly evident that for stroke education campaigns to have any chance of being effective, we need to focus on the disconnect between stroke knowledge and actions. Specifically, we need to understand why stroke patients and their bystanders delay calling emergency medical services.^3,6^ Numerous studies have demonstrated that, contrary to the commonly held premise, increased knowledge of stroke does not translate to an increase in appropriate actions.^9–11^ For public education campaigns to have any hope of modifying OTA times, it is important that they directly increase the motivation to call emergency medical services (9-1-1) quickly. This should be done by targeting outcome expectations, improving stroke recognition skills, and addressing community norms.^3,10^ To increase the motivation to call 9-1-1 immediately after recognizing stroke symptoms, the public must come to believe that acting rapidly will result in better outcomes and that perceived barriers to calling 9-1-1 (such as financial costs and embarrassment) have been removed. Educational efforts should motivate the public to respond quickly to stroke symptoms by connecting rapid response to improved health outcomes. Before investing further public resources in mass education efforts around stroke, we need to return to the drawing board and obtain a much better understanding of the facilitators of and barriers to early and aggressive action among the general public. This greater understanding then needs to inform the development of new educational methods and messages that should be developed with the use of theory-grounded principles and tested with solid evidence-based evaluation methods.^6^ In the absence of these renewed efforts, one wonders if meaningful reductions in the delay between stroke onset and hospital arrival will ever be achievable.
